# Supplementary material for: Empirical methods for controlling false positives and estimating confidence in ChIP-Seq peaks
Source: BMC Bioinformatics. 2008 Dec 5;9:523. doi: 10.1186/1471-2105-9-523 (PMC2628906; doi:10.1186/1471-2105-9-523)
Supplement: Additional File 1 — A variety of html documents from the USeq web site detailing the available applications, their best usage, output file type descriptions, command line menus, etc. [file 1471-2105-9-523-S1.zip › USeqUserGuides/JavaDoc.zip!/JavaDoc/util/bio/parsers/gff/DmelRel4Extractor.html]

DmelRel4Extractor


|  |  |  |
| --- | --- | --- |
| **Overview** \| **Package** \| **Class** \| **Use** \| **Tree** \| **Deprecated** \| **Index** \| **Help** | |  |
| PREV CLASS   **NEXT CLASS** | **FRAMES**    **NO FRAMES**     **All Classes** |  |
| SUMMARY: NESTED \| FIELD \| CONSTR \| METHOD | DETAIL: FIELD \| CONSTR \| METHOD |  |


---


## util.bio.parsers.gff Class DmelRel4Extractor

```
java.lang.Object
  util.bio.parsers.gff.DmelRel4Extractor
```

---

``` public class DmelRel4Extractor extends java.lang.Object ```

Class for building gene models from GFF3Features, specifically tailored to release 4.0 drosophila melanogaster GFF.
The start is always less than the end.
The actual length of an element is end+1-start.
Orientation is 1,-1, or 0 corresponding to +,-, or .

---

**Constructor Summary**

`DmelRel4Extractor()`


| **Method Summary** | |
| --- | --- |
| `void` | `buildGeneGrp(Gff3Feature f)`             Creates a new gene group, call this last after having loaded the exons, transcripts, and translations. |
| `void` | `buildGenericFeature(Gff3Feature f)` |
| `void` | `buildTransGroups()` |
| `void` | `extract(java.io.File gffFile, boolean stripIt)` |
| `java.util.ArrayList` | `getGeneGroupArrayList()` |
| `GeneGroup[]` | `getGeneGroups()`             Final product from the extractor. |
| `java.util.LinkedHashSet` | `getGenericFeatureHash()`             Contains all the feature types that were not recognized by the Extractor. |
| `java.util.ArrayList` | `getGenericFeatures()` |
| `java.util.ArrayList` | `getGenericFeaturesAL()` |
| `java.util.LinkedHashSet` | `getGenericFeaturesHash()` |
| `java.lang.String` | `getRegExGeneTypes()` |
| `boolean` | `isGenericFeaturesFound()` |
| `static void` | `main(java.lang.String[] args)` |
| `void` | `setChromosomeNameAppender(java.lang.String chromosomeNameAppender)` |
| `void` | `setRegExGeneTypes(java.lang.String regExGeneTypes)` |
| `static java.lang.String` | `stripIt(java.util.regex.Pattern patCG, java.util.regex.Pattern patTE, java.util.regex.Pattern patCR, java.lang.String toStrip)` |

**Methods inherited from class java.lang.Object**

`equals, getClass, hashCode, notify, notifyAll, toString, wait, wait, wait`

**Constructor Detail**

### DmelRel4Extractor

```
public DmelRel4Extractor()
```


**Method Detail**

### main

```
public static void main(java.lang.String[] args)
```

---


### extract

```
public void extract(java.io.File gffFile,
                    boolean stripIt)
```

---


### stripIt

```
public static java.lang.String stripIt(java.util.regex.Pattern patCG,
                                       java.util.regex.Pattern patTE,
                                       java.util.regex.Pattern patCR,
                                       java.lang.String toStrip)
```

---


### getGenericFeatureHash

```
public java.util.LinkedHashSet getGenericFeatureHash()
```

:   Contains all the feature types that were not recognized by the Extractor.
    Should be user added items like CRMs, enhancers, etc.

---


### getGenericFeatures

```
public java.util.ArrayList getGenericFeatures()
```

---


### buildGenericFeature

```
public void buildGenericFeature(Gff3Feature f)
```

---


### buildTransGroups

```
public void buildTransGroups()
```

---


### buildGeneGrp

```
public void buildGeneGrp(Gff3Feature f)
```

:   Creates a new gene group, call this last after having loaded the exons, transcripts, and translations.

---


### getGeneGroupArrayList

```
public java.util.ArrayList getGeneGroupArrayList()
```

---


### getGeneGroups

```
public GeneGroup[] getGeneGroups()
```

:   Final product from the extractor. Use in downstream analysis.

---


### isGenericFeaturesFound

```
public boolean isGenericFeaturesFound()
```

---


### getRegExGeneTypes

```
public java.lang.String getRegExGeneTypes()
```

---


### setRegExGeneTypes

```
public void setRegExGeneTypes(java.lang.String regExGeneTypes)
```

---


### getGenericFeaturesAL

```
public java.util.ArrayList getGenericFeaturesAL()
```

---


### getGenericFeaturesHash

```
public java.util.LinkedHashSet getGenericFeaturesHash()
```

---


### setChromosomeNameAppender

```
public void setChromosomeNameAppender(java.lang.String chromosomeNameAppender)
```


---


|  |  |  |
| --- | --- | --- |
| **Overview** \| **Package** \| **Class** \| **Use** \| **Tree** \| **Deprecated** \| **Index** \| **Help** | |  |
| PREV CLASS   **NEXT CLASS** | **FRAMES**    **NO FRAMES**     **All Classes** |  |
| SUMMARY: NESTED \| FIELD \| CONSTR \| METHOD | DETAIL: FIELD \| CONSTR \| METHOD |  |


---
